# Computational Pipeline for the PGV-001 Neoantigen Vaccine Trial

**DOI:** 10.3389/fimmu.2017.01807

**Published:** 2018-01-18

**Authors:** Alex Rubinsteyn, Julia Kodysh, Isaac Hodes, Sebastien Mondet, Bulent Arman Aksoy, John P. Finnigan, Nina Bhardwaj, Jeffrey Hammerbacher

**Affiliations:** ^1^Department of Genetics and Genomic Sciences, Icahn School of Medicine at Mount Sinai, New York, NY, United States; ^2^Department of Microbiology and Immunology, Medical University of South Carolina, Charleston, SC, United States; ^3^Icahn School of Medicine at Mount Sinai, Tisch Cancer Institute, New York, NY, United States

**Keywords:** neoantigens, personalized vaccine, immunoinformatics, genomics, computational pipeline

## Abstract

This paper describes the sequencing protocol and computational pipeline for the PGV-001 personalized vaccine trial. PGV-001 is a therapeutic peptide vaccine targeting neoantigens identified from patient tumor samples. Peptides are selected by a computational pipeline that identifies mutations from tumor/normal exome sequencing and ranks mutant sequences by a combination of predicted Class I MHC affinity and abundance estimated from tumor RNA. The personalized genomic vaccine (PGV) pipeline is modular and consists of independently usable tools and software libraries. We hope that the functionality of these tools may extend beyond the specifics of the PGV-001 trial and enable other research groups in their own neoantigen investigations.

## Introduction

Cancer neoantigens are antigens presented on tumor cells which, due to either mutation or modification, are not presented on normal cells. Neoantigens generated by tumor DNA mutations have been shown to play a significant role in mediating the destruction of tumor cells by the adaptive immune system (1–3). Several groups have used therapeutic vaccines targeting neoantigens to clear tumors in murine models (4–6). Consequently, many human neoantigen vaccine trials are now under way and several have published promising early results (7, 8). Since very few cancer mutations are recurrent between patients, the identification of neoantigens requires a personalized genomic approach (9). We describe the sequencing protocol and immunogenomic pipeline of PGV-001, a neoantigen vaccine trial at the Mount Sinai Hospital (10).

The personalized genomic vaccine (PGV) computational pipeline takes tumor/normal sequencing data as an input and generates a ranked list of mutated peptide sequences. The steps along the way of determining a personalized vaccine’s contents are implemented as configurable independent tools.

## Overview of the PGV-001 Personalized Vaccine Trial

PGV-001 is a phase I clinical trial at Mount Sinai Hospital, studying the safety and immunogenicity of a multipeptide personalized genomic vaccine for the treatment of cancers. A PGV dose consists of 10 synthetic long peptides (11), each containing a somatic mutation from the patient’s tumor, as well as an immunostimulatory adjuvant: poly-ICLC (12). In the PGV-001 trial, the personalized vaccine is administered in the adjuvant setting, for patients who undergo a complete resection and have no evidence of residual disease.

**Figure 1:**
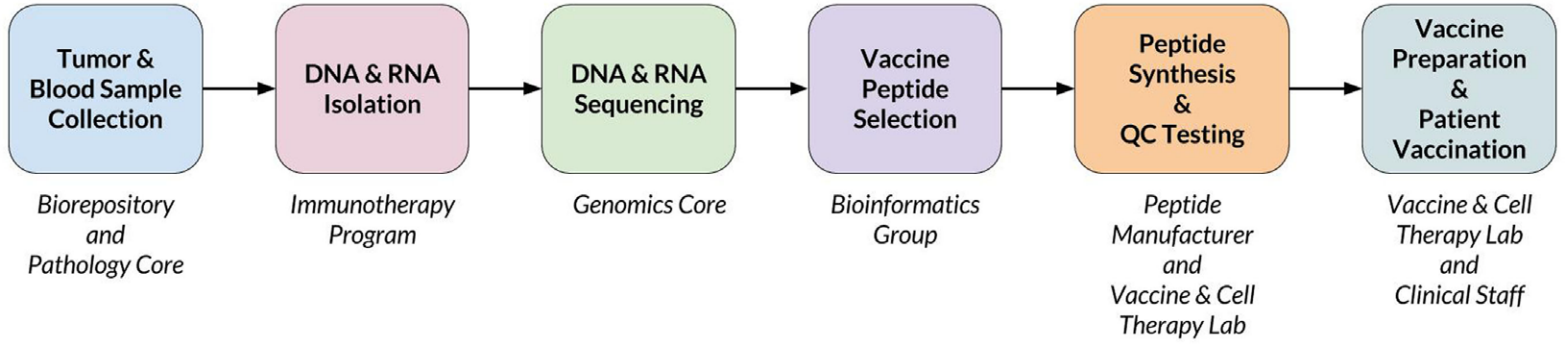
Overview of PGV-001 trial.

**Figure 2:**
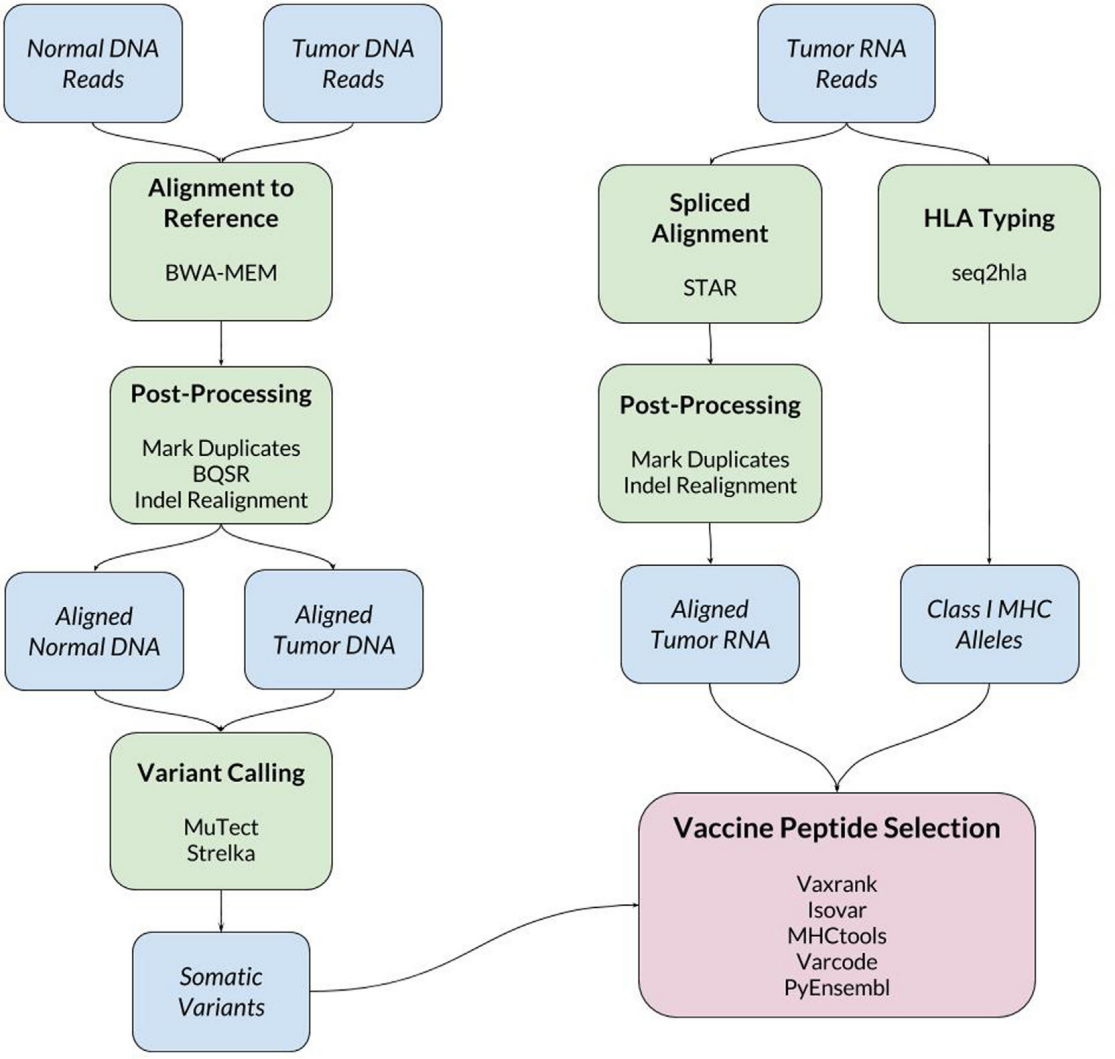
Schematic of bioinformatics tools used in PGV-001 pipeline.

When a new patient enrolls in the trial, their tumor and normal samples are collected and processed to isolate and sequence DNA and RNA. The computational pipeline of PGV-001 is then used to select the peptide contents of the vaccine. The major steps between surgery and vaccination are shown in Figure 1, whereas details of the computational pipeline are shown in Figure 2.

The candidate vaccine peptides generated by the computational pipeline are ranked by abundance and predicted MHC affinity, which both contribute to immunogenicity. The manufacturer attempts to synthesize the top 15 ranked candidate peptide sequences and delivers 10 lyophilized peptides which they are able to purify to sufficient quality and quantity. The peptides are dissolved in DMSO and mixed with poly-ICLC immediately before use. The personalized vaccine is administered as an intracutaneous injection and is given to the patient 10 times over a span of 6 months.

## Sequencing Protocol for DNA and RNA

The sequencing protocols used for both DNA and RNA can dramatically affect the sensitivity of variant detection, and thus ultimately change the results of the vaccine pipeline. The largest determinants of sensitivity are the sample quality, method of sequencing library preparation, and quantity of sequenced reads. Whenever possible, PGV uses fresh frozen tumor tissue samples, which results in significantly improved variant detection accuracy as compared with sequencing of formalin-fixed (FFPE) samples (13). An additional benefit of using fresh frozen samples is that mRNA can be enriched using poly-A capture, whereas the fragmented RNA of FFPE samples can only be prepared with less efficient methods such as ribosomal depletion (14). For patients with solid tumors, normal DNA is extracted from peripheral blood rather than potentially contaminated adjacent tissue (15).

Fragmentation by sonication was preferred to transposase-based methods (16) due to significant sequence bias, leading to lost coverage after marking duplicate reads (17). Among the exome enrichment techniques which use sonication, we chose Agilent’s SureSelect XT kit due to its efficient rate of capturing on-target reads (18).

We chose to target 150× mean coverage for the normal DNA (exome) sequencing since this was found to be the point of diminishing sensitivity across different variant calling pipelines (19). Several of the cancer types allowed in the PGV-001 trial (particularly lung and head/neck cancers) have been shown to result in systematically low purity samples (20). To accommodate a significant degree of non-cancerous DNA, we assume 50% tumor purity and consequently target 300× exome coverage for the tumor DNA sample.

A final consideration is the choice of read length, which does not significantly impact variant discovery from DNA but does impact variant phasing in RNA. Since a 25mer vaccine peptide is translated from 75 bp of coding sequence, PGV could theoretically use any read length above that minimum. To allow for many distinct aligned positions overlapping the same region of coding sequence, the PGV protocol uses 125-bp reads. These provide a good compromise between length and base quality on the HiSeq 2500 instrument.

## Overview of the Computational Pipeline

The inputs to the computational pipeline are unmapped sequencing data from tumor DNA, tumor RNA, and normal patient DNA. The tumor and normal DNA samples are aligned against the human GRCh37 reference genome using BWA-MEM (21). The tumor RNA is aligned using STAR (22), which has been found to have particularly high sensitivity for detecting indel variants (23). Both DNA and RNA alignment files are processed according to GATK Best Practices (24). One noteworthy deviation from the standard GATK pipeline is our use of assembly based indel realignment on tumor RNA data (in addition to the DNA samples). This is done to improve the sensitivity of detecting RNA reads which support indel somatic variants.

### Somatic Variant Calling

Somatic variant calling is performed using MuTect (25) and Strelka (26), whose results are combined by taking a union of called variants. In cases where the final pipeline yields an insufficient number of vaccine peptides (fewer than 15), we rerun the pipeline including MuTect2 among the set of variant callers to increase sensitivity.

### HLA Typing

To make predictions about epitope presentation to T-cells, it is necessary to know the patient’s HLA type. This can be determined computationally from exome or bulk RNA sequencing or validated externally using HLA-specific amplicon sequencing (27). The PGV pipeline currently uses seq2hla (28) for HLA typing from tumor RNA while also using amplicon sequencing of normal DNA for validation. Across 10 patients, the two methods have only disagreed on a single allele, where HLA-C*07:02 was mistyped as HLA-C*07:01. This high degree of concordance matches our previous experience with HLA typing of fresh frozen tissue samples; formalin-fixed tissue is more likely to give discordant results between different sequencing methods.

### Vaccine Peptide Selection

The bulk of the custom software developed for this trial is related to vaccine peptide selection. The results of the above steps are a set of somatic variants, aligned tumor RNA reads, and the patient’s HLA type. These data are then used to determine mutant protein sequences, estimate mutation abundance, predict MHC ligands overlapping mutations, and finally to generate a ranked list of candidate vaccine peptides.

Some of the tools used in vaccine peptide selection include:

- *Vaxrank* (29): overall vaccine selection tool with ranking logic.
- *Isovar* (30): determines mutant protein sequence from somatic variants and tumor RNA.
- *Varcode* (31): predicts variant effects for filtering out silent mutations.
- *PyEnsembl* (32): provides reference genome annotations that are used by Varcode to determine exon boundaries and transcript sequences.
- *MHCtools* (33): common interface to peptide–MHC-binding predictors.

Due to their importance, Isovar and Vaxrank are both described in greater detail in the following two sections.

## Isovar: Determining the Mutant Protein Sequence

There are several different software packages that predict the protein-level effect of a coding mutation (31, 34, 35). However, for the purposes of selecting a vaccine peptide’s sequence, it is not sufficient to predict a DNA mutation’s protein effect without considering the transcripts in which it occurs. A somatic mutation can be associated with selective splicing of particular RNA isoforms (36) and can also cooccur with other genomic variants. Thus, in the PGV pipeline, the tumor RNA sequencing data are also used to determine a mutant coding sequence.

**Figure 3:**
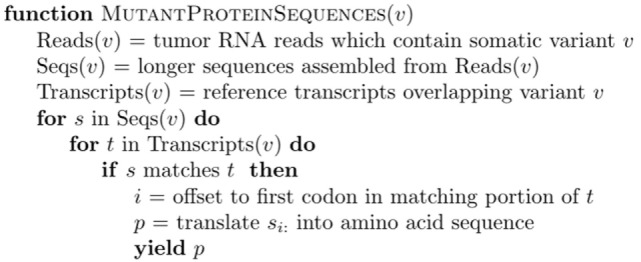
Overview of Isovar algorithm for determining mutant protein sequences.

For each mutation, it is possible to infer multiple coding sequences from supporting RNA reads due to sequencing error, splicing diversity, and tumor heterogeneity. To account for these potentially complicating factors, we developed a tool called Isovar (30), which can be downloaded from https://github.com/hammerlab/isovar. Isovar uses RNA to assemble the most abundant coding sequence for each mutation. An overview of the algorithm is given in Figure 3.

**Figure 4:**
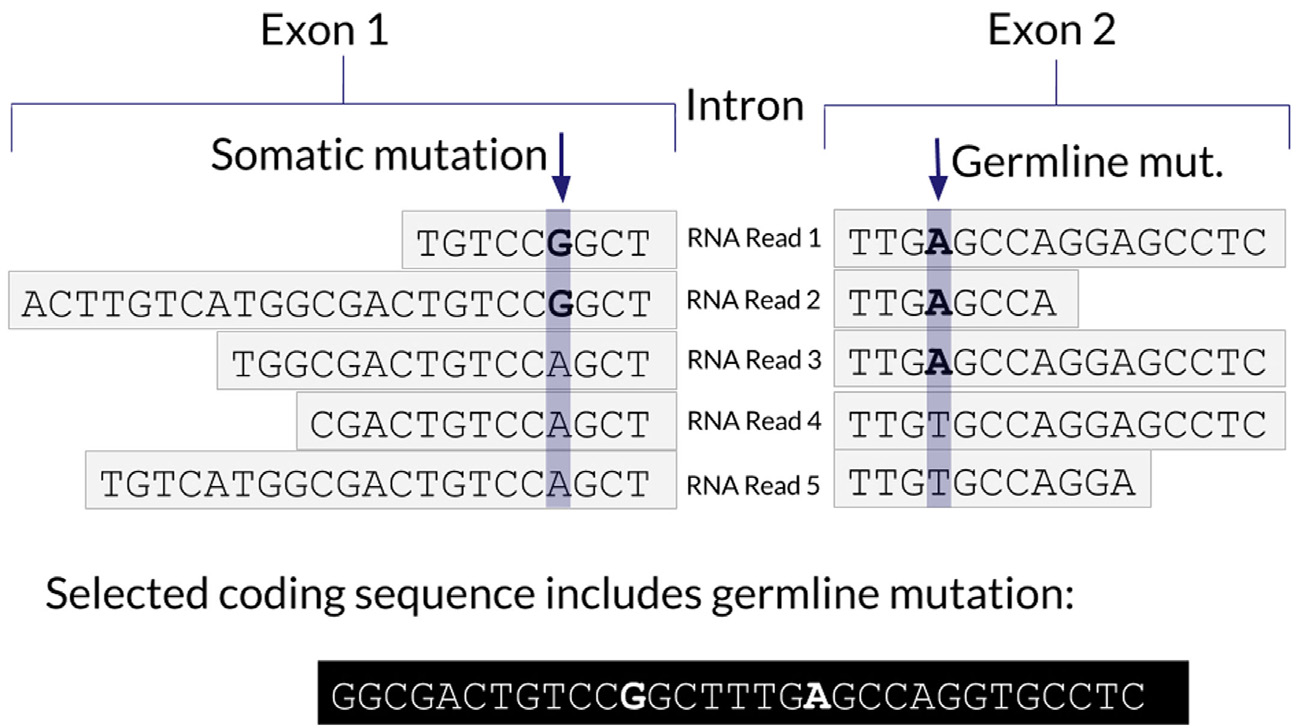
Schematic representation of a somatic mutation co-occurring with a germline mutation.

**Figure 5:**
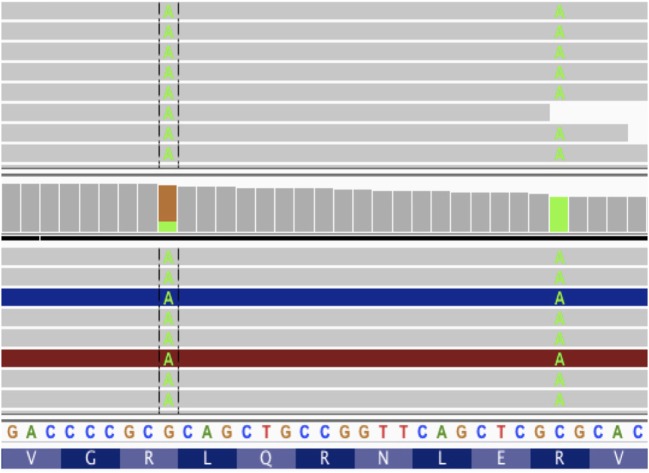
Screenshot from IGV with tumor DNA on top and tumor RNA on bottom. The two somatic variants from patient data 7 amino acids apart. If these mutations were considered without phasing, we would get two different vaccine peptides, neither of which would match the protein sequence produced by tumor cells.

One advantage of using RNA to determine the coding sequence is that it phases adjacent (germline or somatic) variants. Examples of the impact of adjacent variants on a coding sequence are shown in Figures 4 and 5. A further advantage is that Isovar, by using mutation-supporting RNA reads to determine each mutant protein sequence, naturally estimates allele-specific expression. If the PGV pipeline, on the other hand, used bulk expression it would potentially overestimate how much of a mutant protein is being made. In an extreme case, all of the RNA reads aligning to a particular gene could be wild type, with none supporting the somatic variant of interest.

## Vaxrank: Vaccine Peptide Selection

Once we have determined the amino acid sequences containing somatic mutations and estimated their abundance in the tumor, the final step is to rank them according to desirability of inclusion in personalized vaccine.

There are many potential correlates of immunogenicity that can be used to prioritize neoantigens, such as expression, MHC-binding affinity, peptide–MHC complex stability, proteasomal cleavage, and other antigen-processing steps. Of those, the PGV pipeline optimizes for high expression and predicted strong Class I MHC binding. There are several published computational predictors of Class I MHC-binding affinity which have demonstrated high accuracy (37–39). PGV uses NetMHCpan (37) due to its extensive coverage of patient alleles.

**Figure 6:**
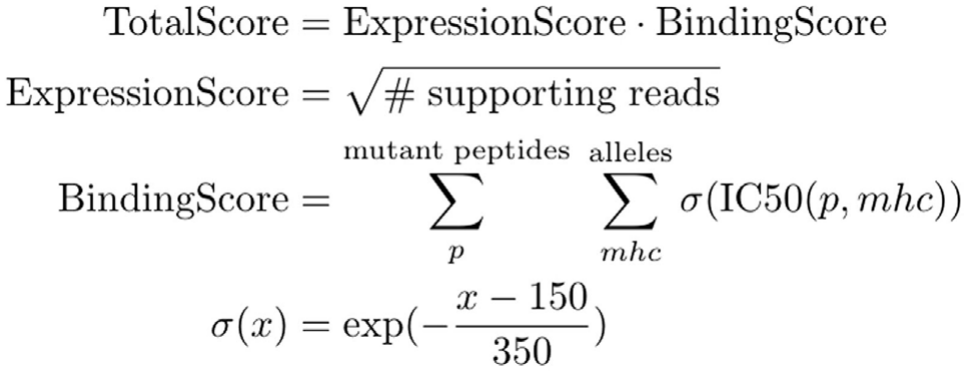
TotalScore used to rank somatic variants in a way that attempts to balance predict MHC binding and abundance. ExpressionScore uses read count (instead of a normalized measure like FPKM) since these scoring criteria are not meant to be compared between patient samples. BindingScore sums normalized binding affinities of mutant peptides across all patient alleles and lengths between 8 and 11.

The final ranking of candidate vaccine peptides according to predicted MHC binding and expression is performed by a tool called Vaxrank (29). Vaxrank identifies high-affinity mutant MHC ligands within each peptide and combines these predictions into a single MHC-binding score. This score is then scaled according to the expression of that mutation in the tumor. The formula for computing these MHC and expression scores is given in Figure 6. The scale and offset for MHC affinity normalization was determined by a logistic fit of affinity versus immunogenicity from the dataset used to determine the classical 500-nM affinity threshold (40). There is no rigorous justification for the multiplicative scoring function, other than the intuition that epitope abundance and MHC affinity are independent prerequisites for immunogenicity.

Since some peptides cannot be manufactured using solid-phase synthesis, our vaccine peptide selection algorithm includes manufacturability heuristics, such as minimization of cysteine content.

Vaxrank can be downloaded from https://github.com/hammerlab/vaxrank.

## Epidisco: Parallel Implementation of the PGV Pipeline

The workflow management tool that orchestrates the execution of the PGV pipeline is called Epidisco (41). Epidisco is used to set up a local or cluster compute environment, install all relevant bioinformatics tools (external software such as GATK, as well as our own tools including Isovar and Vaxrank), and coordinate the execution of these different tools on the input data.

Epidisco accelerates portions of the genomics pipeline on two levels. Independent computational tasks such as QC checks, the processing of the RNA-sequencing data, and the joint analyses of the normal-tumor DNA-sequencing data are all run in parallel. Within the invocation of each tool, when possible, sequencing data are split into multiple segments, partial results are computed in parallel and then merged.

Epidisco supports local computation, traditional HPC schedulers such as LSF, and cloud-based resources from Google Cloud and AWS. On a typical machine, running the complete PGV pipeline for a single patient can take up to 4 days; but making use of five or more computers for parallelization reduces the overall running time down to a single day.

Epidisco also makes the PGV pipeline tolerant to failures of intermediate steps and allows resuming the pipeline from the point of failure with a simple restart request. By handling such failures in an automated way, carrying out cleaning procedures, and restarting only the tasks that need to be rerun, the workflow makes it easier for researchers to operate such complex computational tasks. Epidisco provides command line and web-based utilities to facilitate starting a new workflow, collecting the results, and troubleshooting specific parts of a pipeline.

The individual infrastructure tools used by the PGV pipeline are implemented as an OCaml stack and include:

- *Ketrew*: custom workflow manager who handles dependency management, parallelization, and smart restarts of failed tasks.
- *Biokepi*: wraps bioinformatics tools so they can be used with Ketrew and statically ensures the absence of common mistakes during pipeline construction.
- *Secotrec*: cluster management tool that allows deployment on cloud services such as the Google Cloud Platform and Amazon Web Services.
- *Epidisco*: the actual implementation of the PGV-001 pipeline.

## Discussion

The PGV pipeline is a modular, highly configurable, freely available method for selecting the contents of a therapeutic neoantigen vaccine. The PGV pipeline has been used to predict vaccine peptides for several mouse models (LLC, B16 F1/F10), five “dry run” patients whose samples were processed according to the PGV protocol but did not participate in the trial, and five patients who were being considered for enrollment in the trial. Of the patients eligible for enrollment, one has been treated so far and another enrolled. The remainder did not enroll due to progression of disease or low-quality tumor samples.

Several other groups have released pipelines for neoantigen vaccine prediction, most notably pVAC-seq (42) and MuPeXI (43). A deep comparison between neoantigen pipelines likely requires evaluating T-cell response and antitumor activity after vaccination, which is beyond the scope of this paper. There are, however, a few obvious differences between the PGV pipeline and others which have been published:

- *Modularity*: the PGV pipeline has been developed as a collection of flexible standalone tools, rather than a single monolithic script. These tools can be repurposed for other immunogenomics analyses and have already been used for retrospective analyses of checkpoint blockade clinical trials (44).
- *Inputs are FASTQ files*: MuPeXI and pVAC-seq both require the implementation of separate genomics pipeline to infer patient HLA type, call somatic variants, and quantify expression. The PGV pipeline, by contrast, is self-contained in the sense that its inputs are raw FASTQ files and its outputs are vaccine peptide predictions.
- *Dependence on tumor RNA*: the PGV pipeline relies on tumor RNA reads to determine the mutant protein coding sequence. MuPeXI and pVAC-seq, by contrast, only consider expression data after predicting a mutant protein sequence from a variant in isolation. PGV’s approach has potential benefits in capturing altered patterns of splicing and phasing somatic variants with other nearby variants. These potential benefits, however, have yet to be evaluated systematically.
- *Liberal software license*: all of the software components that comprise the PGV pipeline are freely available under the Apache software license. MuPeXI does not yet appear to have a fully open source license, while pVAC-seq uses the more restrictive non-profit open software license.
- *Optimization of peptide sequence for solid-phase synthesis*: PGV appears to be unique among freely available neoantigen pipelines in attempting to choose peptides whose sequence content is more likely to be successfully manufactured. We have found this to be an important step, especially when using longer peptides, due to the significant delays introduced by failed synthesis or purification attempts.

The PGV-001 trial is the first in a series of planned neoantigen vaccine investigations. Several improvements to the PGV pipeline are planned, including the use of genomic fusions and other structural variants as neoantigen sources, clonality as a consideration for variant prioritization, and additional immunological predictions such as proteasomal cleavage and Class II MHC binding. As immune response data from ongoing preclinical work and PGV-001 becomes available, our method for combining correlates of immunogenicity into a single ranking will require extensive evaluation.

## Author Contributions

The first draft of this paper was originally written by IH and substantially revised by AR and JK. Genomics and immuno-informatics tools were implemented by AR, JK, and other lab members. The workflow libraries were written mostly by SM. The actual pipeline (connecting all the individual tools and running them in parallel) was written mostly by IH with help from BA. The overall design of the software was determined through extensive conversation with JH. The PGV trial was designed by NB, JF, AR, and several others in the Bhardwaj lab.

## Conflict of Interest Statement

The authors declare that the research was conducted in the absence of any commercial or financial relationships that could be construed as a potential conflict of interest. The reviewer BS and handling editor declared their shared affiliation.
